# Unique Presentation of *Mycoplasma pneumoniae*-Induced Rash and Mucositis with Salivary Gland Inflammation in a Pediatric Patient: A Case Report

**DOI:** 10.3390/jcm13164587

**Published:** 2024-08-06

**Authors:** Izabela Kucharek, Klaudia Bednarz, Adam Jerzy Sybilski

**Affiliations:** Clinical Department of Pediatrics and Allergology, National Medical Institute of the Ministry of the Interior and Administration, 02-507 Warsaw, Poland; izabela.orlinska@cskmswia.gov.pl (I.K.); adam.sybilski@cskmswia.gov.pl (A.J.S.)

**Keywords:** *Mycoplasma pneumoniae*, atypical respiratory pathogen, community-acquired pneumonia, salivary gland inflammation, mucositis

## Abstract

**Background:** *Mycoplasma pneumoniae* (MP) is a significant respiratory pathogen leading to community-acquired pneumonia (CAP), especially in children. Up to 30% of confirmed MP cases can develop dermatological symptoms, with *Mycoplasma pneumoniae*-induced rash and mucositis (MIRM) being a distinct clinical entity marked by mucous membrane inflammation, with or without skin lesions. **Methods**: This case report describes a 7-year-old girl admitted with fever, stomatitis, conjunctivitis, and skin lesions. On the second day, a painful neck enlargement was observed, with ultrasound confirming bilateral submandibular salivary gland inflammation and elevated serum amylase levels. The patient later developed pneumonia, confirmed via chest X-ray and pleural ultrasound. MP infection was confirmed via specific IgM antibodies and PCR in a throat swab. **Results:** The patient was diagnosed with MIRM and was treated with clarithromycin, amoxicillin with clavulanic acid, and methylprednisolone, resulting in a full recovery. **Conclusions:** This case highlights a unique presentation of MIRM with salivary gland inflammation, not previously described in pediatric mycoplasmal infections.

## 1. Introduction

*Mycoplasma pneumoniae* (MP) is a common respiratory pathogen that can lead to community-acquired pneumonia (CAP), especially in children. Epidemiological studies have shown that dermatological manifestations of *Mycoplasma pneumoniae* infection, such as rashes, occur in about 10–30% of patients with confirmed infection [1,2]. In children, the incidence of these symptoms is higher than in adults [2]. *Mycoplasma pneumoniae*-induced rash and mucositis (MIRM) has now been recognized as a distinct clinical entity. The condition is characterized by marked inflammation of the mucous membranes with or without skin lesions and was first systematically described and differentiated from the erythema multiforme (EM)/Stevens–Johnson syndrome (SJS)/toxic epidermal necrosis (TEN) spectrum by Canavan et al. in 2014 due to its unique clinical picture and usually milder course of disease [3]. Mucocutaneous lesions similar to MIRM have also been described in the course of infections caused by Chlamydophila pneumoniae, influenza B virus, enterovirus/rinovirus, human metapneumovirus, human parainfluenza virus 2, adenovirus, and SARS-CoV-2 [4,5,6]. This has led to the proposal of a new term to describe these conditions—Reactive Infectious Mucocutaneous Eruption (RIME). The term RIME, introduced by the Pediatric Dermatology Research Alliance (PeDRA) in 2018, unifies the nomenclature for such reactions, which can be caused by a variety of pathogens, including *Mycoplasma pneumoniae* [7].

The pathophysiology of MIRM is not fully understood but is thought to involve immune mechanisms such as B-cell proliferation, deposition of immune complexes, and molecular mimicry between Mycoplasma adhesion molecules and keratinocyte antigens [8,9]. Clinically, MIRM manifests as a widespread inflammation of the mucous membranes affecting the mouth, eyes, and urogenital areas and may include a variety of skin lesions. The mucosal lesions can be painful and significantly affect the patient’s quality of life [9]. In the next section, we present a new case of MIRM in a young patient who developed salivary gland inflammation, a symptom not previously described in the literature in either adults or children. The authors hope that describing this case will help to diagnose MIRM more quickly and introduce appropriate treatment in other patients with similar symptoms.

## 2. Case Report

A generally healthy 7-year-old girl was admitted to the hospital due to dehydration in the course of fever and stomatitis accompanied by conjunctivitis and skin lesions. A cough had persisted for a week before admission, a runny nose and signs of conjunctivitis for 4 days, and a fever of up to 39 °C for 3 days. Two days before hospitalization, the girl began to complain of a sore throat and difficulty swallowing. On the day before admission, a non-itchy erythematous-pigmented rash appeared on the face, trunk, and lower extremities, as well as erosions in the mouth. The child was apathetic, refusing to eat and drink. There were no features of infection in the rest of the household.

**Figure 1:**
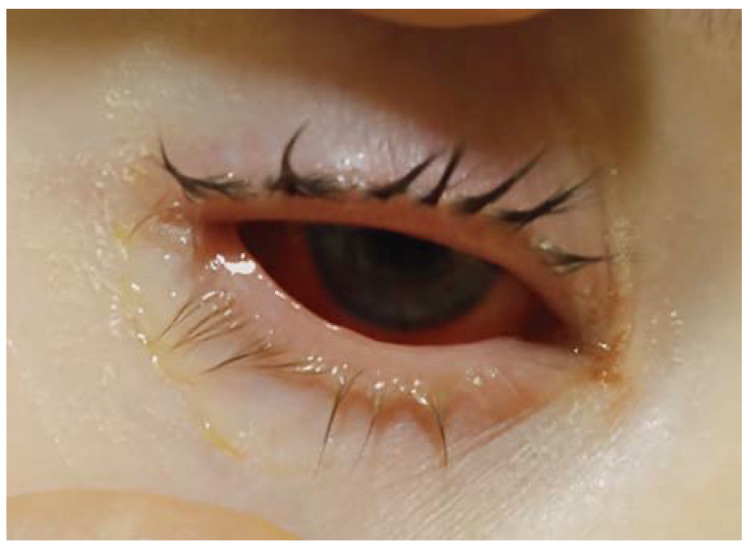
Conjunctiva of the left eye with purulent discharge sticking the eyelashes together and making it difficult to open the eyelid.

**Figure 2:**
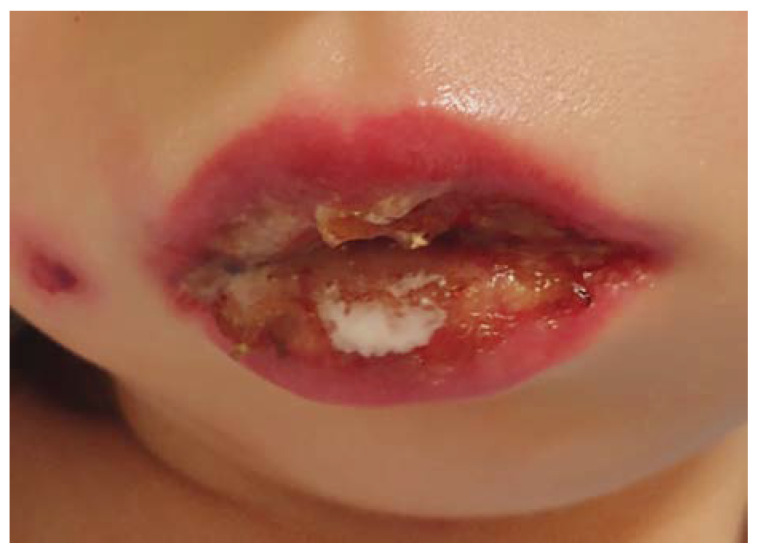
Edema, erosions, and scabs of the red lips and a skin lesion with visible central ulceration, a zone of edema, and erythema on the right cheek.

On admission, the girl was in an average general condition. There were isolated erythematous and papular lesions on the skin of the face, trunk, and lower extremities. There were features of bilateral conjunctivitis—congestion with profuse mucopurulent discharge in the conjunctival sacs, as well as swollen eyelids, making it difficult to open the eyes—and photophobia (Figure 1). The labial redness was swollen with erosions and scabs (Figure 2). Numerous erosions and thrush were present on the oral mucous membranes. The tonsils were enlarged and covered with white plaque. Auscultation showed no abnormalities.

Laboratory results showed elevated acute phase protein levels of 46.4 mg/L with normal leukocyte levels of 6960/mm^3^.

Treatment included acyclovir, ofloxacin, intravitreal corticosteroids, and nystatin for oral lesions. The patient required regular ophthalmologic follow-up to clear the conjunctiva of pseudomembranes.

**Figure 3:**
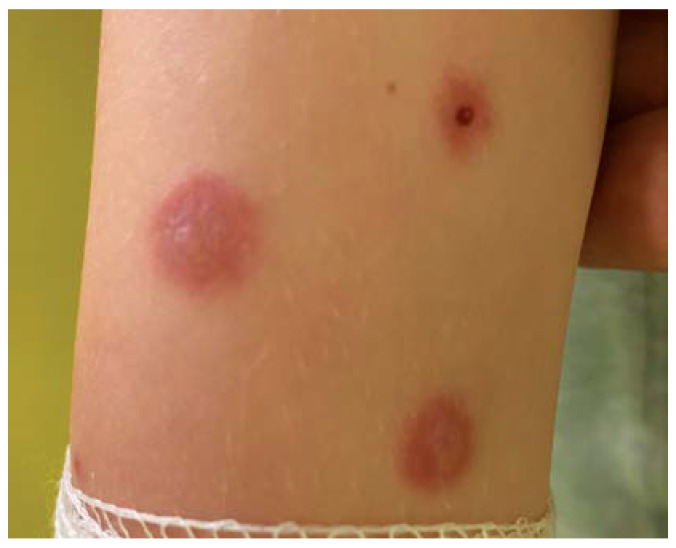
Shotgun disc-like lesions on the left arm.

On the next day of hospitalization, a painful enlargement of the neck circumference was observed and ultrasound showed features of bilateral submandibular salivary gland inflammation. Serum amylase levels were elevated at 378 U/L. There were also isolated new lesions on the skin with the character of shooting disc-erythematous lesions with a flaccid blister in the central part (Figure 3). Dark discoloration in the center was observed on the previously present lesions.

**Figure 4:**
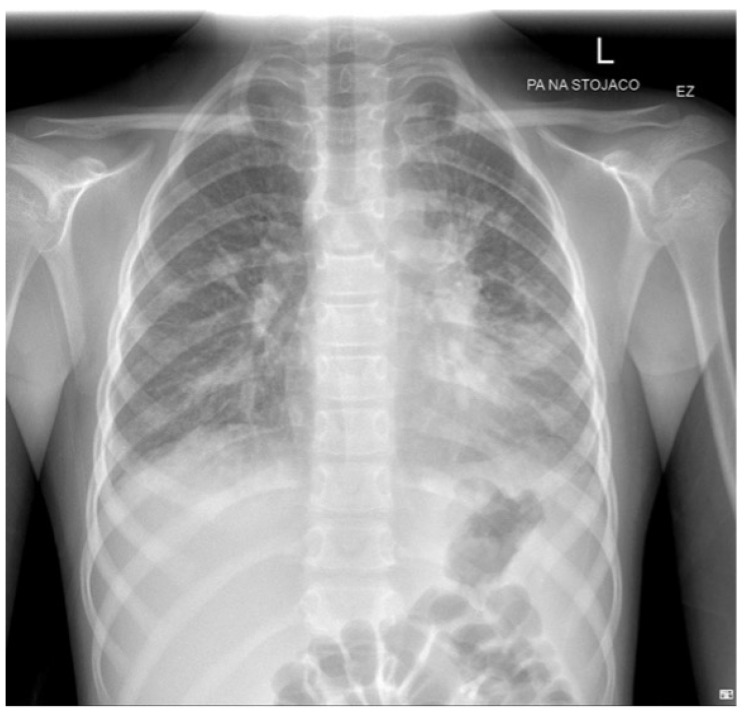
Chest X-ray: parenchymal and interstitial thickening within both lung fields spreading distally, most severe at the base of the left lung.

On the third day of hospitalization, features of dyspnea were observed—respiratory rate accelerated to 30/min. Auscultation revealed a muted alveolar murmur at the base of both lungs. Chest X-ray described parenchymal–interstitial thickening within both lung fields spreading distally, most severe at the base of the left lung, and ultrasound of the pleural cavities showed fluid bilaterally (Figure 4).

A positive result of specific antibodies against *Mycoplasma pneumoniae* in the IgM class was obtained. Infection was also confirmed via PCR in a throat swab.

Antibiotic therapy was administered with clarithromycin (15 mg/kg/day for 10 days) and due to the extent and nature of the radiological lesions, additionally with amoxicillin with clavulanic acid (90 mg of amoxicillin/kg/day for 7 days). Methylprednisolone was added to the treatment (1 mg/kg/day for 7 days).

During the following days of hospitalization, a gradual improvement in the general condition was observed, as well as the resolution of the swelling of the salivary glands, skin, and mucosal lesions. In additional examinations, a decrease in inflammatory exponents was observed, as well as a decrease in inflammatory consolidations and the amount of fluid in the pleural cavities in control lung ultrasound.

After 14 days of hospitalization, the patient was discharged in a good general condition. The results of laboratory tests normalized, as shown in Supplementary Table S1. Due to decreased visual acuity, it was recommended to continue local antibiotic therapy for 5 days, as well as steroid therapy. The girl was referred for further outpatient ophthalmological care.

## 3. Discussion

M. pneumoniae is estimated to cause up to 40% of out-of-hospital pneumonia cases and 18–19% of pneumonia in children that require hospitalization [10,11]. MIRM (*Mycoplasma pneumoniae*-induced rash and mucositis) was first defined in 2015 as a mucocutaneous syndrome occurring in the context of pneumonia caused by M. pneumoniae. MIRM most commonly affects children and adolescents, especially boys, and usually follows a prodromal phase of respiratory symptoms such as fever, malaise, and cough that precedes dermatological symptoms by an average of two days [12]. The proposed diagnostic criteria for MIRM are listed in Table 1 [3].

The skin lesions described in MIRM are usually sparse and consist mainly of bullous and disc-like lesions concentrated on the extremities [12]. Mucositis is inherent in MIRM, with involvement of the oral cavity in 94% of cases, the eyes in 82–87%, and the genitourinary tract in 63%. As in our patient’s case, oral mucositis is often the main indication for hospitalization and the cause of morbidity [3]. MIRM can be easily confused with infection caused by herpes simplex virus (HSV). This is due to similarities in the clinical presentation of the two conditions, especially in terms of cutaneous and mucosal manifestations. Both cases may present with blisters and erosions on the oral, ocular, and genital mucous membranes, leading to potential diagnostic confusion and the inclusion of acyclovir as a first-line treatment [12]. A correct diagnosis is based mainly on the clinical picture supported by serological and radiological evidence of MP infection. In the case cited above, salivary gland inflammation was diagnosed on the sixth day of illness, based on clinical, ultrasound, and laboratory signs (elevated serum amylase levels), while ruling out other possible infectious or mechanical causes of the condition. Salivary gland inflammation can be caused by a number of pathogens, most commonly by viruses such as mumps virus, influenza viruses, and parainfluenza viruses. Among the bacteria most commonly responsible for salivary gland inflammation is Staphylococcus aureus. Less commonly, inflammation can be caused by other bacteria such as Streptococcus pneumoniae [13]. The only available case report of salivary gland inflammation in the course of a mycoplasmal infection dates back to 1980. The patient described in it presented mucositis and pneumonia in addition [14].

Treatment of MIRM has not yet been studied in controlled trials, and there are currently no published treatment guidelines. The most common treatment is antibiotics directed against M. pneumoniae in combination with pain management, intravenous hydration, and mucosal care [8]. Case reports and small case series have described the use of corticosteroids, intravenous immunoglobulins, and cyclosporine [15,16]. Overall, MIRM has a benign course, with less than 5% of patients requiring an intensive care unit and 81% of patients being completely cured [3]. Ophthalmic complications, including corneal ulceration and blindness, are the most common long-term consequences, occurring in about 9% of patients [8].

## 4. Conclusions

This article describes the case of a 7-year-old girl with *Mycoplasma pneumoniae*-induced rash and mucositis (MIRM), characterized by the unusual manifestation of salivary gland inflammation, which has not yet been reported in the literature in the context of MIRM. This case report underscores the need to include salivary gland inflammation in the diagnosis of MIRM and highlights the need for further research to better understand this rare but important clinical manifestation.

## Figures and Tables

**Table 1:** Diagnostic criteria for *Mycoplasma pneumoniae*-induced rash and mucositis (MIRM).

Diagnostic Criteria	Description
Minimal skin involvement	<10% of the total body surface area. Up to one-third of cases may have no skin lesions.
Vesiculobullous or diffuse atypical thyroid lesions	The rash can be scattered all over the body, often concentrated on the trunk, extremities, and face. Blisters may burst, leading to erosions and scabs. The eruptions can be painful and itchy.
Involvement of two or more mucosal sites	Includes inflammation of the mucous membranes of the mouth, conjunctiva, and genitals.
Clinical and laboratory evidence of atypical pneumonia	Clinical: fever, cough, auscultatory changes.
	Laboratory: increase in IgM MP antibodies, MP in oropharyngeal or follicular cultures, PCR.

Legend: MP—*Mycoplasma pneumoniae*; PCR—polymerase chain reaction (PCR).

## Data Availability

The original contributions presented in the study are included in the article (and Supplementary Materials), further inquiries can be directed to the corresponding authors.

## Supplementary Materials

The following supporting information can be downloaded at: https://www.mdpi.com/article/10.3390/jcm13164587/s1, Table S1: Laboratory results.
