# Supplementary material for: Unique Presentation of Mycoplasma pneumoniae-Induced Rash and Mucositis with Salivary Gland Inflammation in a Pediatric Patient: A Case Report
Source: J Clin Med. 2024 Aug 6;13(16):4587. doi: 10.3390/jcm13164587 (PMC11354799; doi:10.3390/jcm13164587)
Supplement: Supplementary file 1 [file jcm-13-04587-s001.zip › jcm-3124843-supplementary.pdf]

**Supplementary Table S1. Laboratory Results**

| Parameter | Day 1 | Day 2 | Day 3 | Day 4 | Day 5 | Day 6 | Day 8 | Day 14 |
|---|---|---|---|---|---|---|---|---|
| Leukocytes (WBC) (4500-13,500/mm$^3$) | 6960 | - | - | 5550 | 5410 | 7570 | 12,020 | 11,920 |
| Erythrocytes (RBC) (4.0-5.2 million/mm$^3$) | 4.82 | - | - | 4.62 | 4.78 | 4.9 | 5.51 | 5.1 |
| Hemoglobin (HGB) (11.9-14.7 g/dL) | 12.6 | - | - | 12.1 | 12.3 | 12.7 | 14.6 | 13.5 |
| Platelets (PLT) (150-400 × 10$^9$/L) | 282 | - | 320 | 357 | 455 | 480 | 647 | 472 |
| C-Reactive Protein (CRP) (<5 mg/L) | 46.4 | - | 34.1 | 30.2 | 19.4 | 8.2 | 4.6 | 0.7 |
| Procalcitonin (<0.5 ng/ml) | 0.45 | - | 0.13 | 0.1 | 0.07 | - | - | - |
| Alanine Transaminase [U/l] (<39) | 11 | - | - | 9 | 9 | 5 | - | - |
| Aspartate Transaminase [U/l] (<47) | 34 | - | 21 | 18 | 15 | - | - | - |
| Albumin [g/dL] (3.8-5.4) | 4.21 | 3.26 | - | 3.07 | 3.67 | 3.27 | 4.83 | 4.09 |
| Total Amylase [U/l] (28-100) | 378 | - | 160 | - | - | - | 74 | - |
| Glucose [mg/dL] (70-99) | 79 | - | - | 123 | 87 | 82 | 185 | 76 |
| Creatinine (0.4-0.87 mg/dL) | 0.47 | - | 0.31 | 0.29 | 0.27 | 0.29 | 0.45 | - |
| Urea (17-49 mg/dL) | 43 | - | - | 19 | 35 | 27 | - | - |
| Sodium (132-145 mmol/L) | 136 | - | - | 140 | 139 | 139 | 142 | 139 |
| Potassium (3.1-5.1 mmol/L) | 4.36 | - | - | 3.4 | 4.7 | 4.12 | 4.2 | 4.76 |

* normal range in parentheses
